# Educational Strategy for the Development of Musculoskeletal Competencies in Therapeutic Exercise Through Service-Learning in Community Spaces: A Pilot Study

**DOI:** 10.3390/muscles4030021

**Published:** 2025-07-03

**Authors:** Alejandro Caña-Pino, María Dolores Apolo-Arenas

**Affiliations:** 1 Surgical Medical-Therapy Department, Medicine Faculty and Health Sciences, University of Extremadura, 06006 Badajoz, Spain; mdapolo@unex.es; 2 Research Group PhysioH (Fisioterapia e Hipoterapia), University of Extremadura, 06006 Badajoz, Spain

**Keywords:** service-learning, physiotherapy education, competency-based education, community-based exercise, cardiorespiratory rehabilitation, musculoskeletal education, muscle strength, therapeutic exercise

## Abstract

Service-Learning (SL) is an innovative educational methodology that integrates academic learning with active community engagement, fostering both technical and transversal competencies. This pilot study explores the implementation of an SL-based experience within the Physiotherapy Degree at the University of Extremadura. The primary objective was to design and deliver therapeutic exercise programs targeting patients with cardiorespiratory conditions, utilizing local community resources. A total of 44 third-year physiotherapy students participated in the design and simulated the implementation of community-based interventions targeting muscular strength, postural control, balance, and endurance. A mixed-methods approach was used, combining descriptive statistics (SPSS v23) and thematic analysis of student reflections to assess the impact of SL on the development of specific professional competencies, including clinical reasoning, patient communication, therapeutic planning, and adaptation of interventions to diverse environments. The results show a significant improvement in students’ theoretical and practical understanding, with over 70% of participants rating their learning experience between 8 and 10 (on a 0–10 scale) in aspects such as pathology description, clinical assessment, and exercise planning. Additionally, 92% reported improved teamwork, 89% noted better adaptability, and 87% reported enhanced decision-making skills. The findings suggest that SL can enhance perceived learning in musculoskeletal rehabilitation and support the transition from academic training to clinical practice. However, the study is exploratory and based on perceived outcomes, and future research should include validated tools and real patients to assess its impact more rigorously. This pilot study highlights the value of integrating musculoskeletal-focused training—targeting strength, balance, and endurance—into physiotherapy education through Service-Learning methodology. The study highlights SL’s potential to enrich physiotherapy education while leveraging community spaces—such as those in Extremadura, a region with three UNESCO World Heritage Sites—as dynamic learning environments.

## 1. Introduction

The university education system faces the ongoing challenge of preparing professionals who are not only equipped with specialized technical knowledge but also capable of demonstrating transversal competencies that allow them to adapt to dynamic and diverse professional contexts [1]. In this regard, Service-Learning (SL) has emerged as an effective active-learning methodology that combines academic instruction with community engagement, fostering the development of competencies such as critical thinking, leadership, and problem-solving [2].

In the field of Health Sciences, and particularly in Physiotherapy, SL serves as a valuable pedagogical strategy. It provides students with the opportunity to apply theoretical knowledge in real-world scenarios, thereby enhancing their skills in clinical reasoning, patient-centered communication, and community intervention [3]. Moreover, SL encourages interprofessional collaboration, promoting a holistic and multidimensional approach to patient care [4].

Moreover, physiotherapy students engaged in these learning experiences not only acquire theoretical and procedural skills, but also gain deeper insights into the role of muscles in postural control, mobility, and cardiopulmonary efficiency. By focusing on muscular adaptations, exercise tolerance, and biomechanical load, students enhance their understanding of how to tailor interventions to individual patient needs. This pedagogical approach supports the development of a clinical mindset centered on musculoskeletal health as a fundamental component of holistic physiotherapy care. For instance, randomized trials in similar educational settings have observed that students who implement exercise programs for patients with cardiorespiratory conditions—including strength and endurance training—demonstrated improvements in both clinical knowledge and application skills [3,5]. In addition, simulation-based interventions using standardized or peer patients have been shown to enhance students’ muscle function awareness, movement control, and readiness to apply musculoskeletal assessments in real clinical environments [6].

The utilization of public and community spaces as therapeutic environments has gained increasing relevance. These spaces offer accessible and sustainable alternatives for managing various health conditions, such as in older adults [3,7,8], heart transplant recipients [3,5], and individuals with severe mental disorders [1]. Nevertheless, undergraduate training continues to face challenges in incorporating strategies that support the practical application of theoretical knowledge in real-life contexts. Designing experiential learning activities in such environments allows students to assess the accessibility, opportunities, and limitations of the resources available [9]. Recent systematic reviews confirm SL’s effectiveness in health professions education [10,11]. In physiotherapy, SL enhances clinical decision-making, empathy, and civic engagement.

An additional preparatory step that can significantly contribute to the success of community-based interventions is having students personally experience and reflect on their own proposed therapeutic activities. This immersive process helps them identify strengths, limitations, and potential implementation barriers, improving their ability to adapt interventions effectively for specific patient populations and optimizing the use of available resources. Reflective practice, grounded in experiential learning theory, has been shown to enhance students’ professional development and clinical reasoning skills when explicitly taught and facilitated [12]. Studies in physiotherapy education further confirm that guided reflection during community and clinical placements leads to greater self-awareness, adaptability, and readiness for professional practice [13,14].

Numerous studies have underscored the benefits of SL in health professional education. Arena et al. [15] reported that physiotherapy students participating in SL initiatives experienced increased clinical confidence, improved communication abilities, and heightened empathy toward patients. Similarly, Borstad et al. [16] found that exposure to community-based settings through SL enhanced students’ decision-making and real-world problem-solving skills. Other research has highlighted that SL not only reinforces theoretical and practical learning, but also strengthens community engagement and readiness for clinical practice [17].

Additionally, Noonan et al. [18] emphasize the positive influence of SL on pain management and chronic disease care, suggesting that it cultivates greater sensitivity to individual patient needs. Coffin et al. [19] point to the relevance of SL in interprofessional education, as it enables collaboration between students from diverse disciplines, fostering a more integrated and team-based approach to healthcare.

In recent years, therapeutic exercise has been recognized as a key intervention for improving musculoskeletal function in both healthy and clinical populations. In the context of cardiorespiratory rehabilitation, the strengthening of specific muscle groups such as the respiratory muscles, core stabilizers, and lower limb extensors plays a critical role in restoring functional capacity and improving quality of life. For example, respiratory and core muscle training has been shown to improve walking function, balance, pulmonary performance, and quality of life in post-stroke patients, while systematic reviews demonstrate that respiratory muscle training significantly enhances exercise tolerance, pulmonary function, and strength in individuals with stroke or other cardiorespiratory disorders [20,21]. Community-based pulmonary rehabilitation, which integrates physical training with functional muscle exercises, is now recognized as being integral to COPD and interstitial lung disease care, and has been shown to enhance musculoskeletal and cardiovascular outcomes in real-world settings. Service-Learning projects that incorporate physical training in community environments like parks or sports centers offer an innovative way to bridge academic learning with hands-on experience in muscle rehabilitation. These settings enable the design of functional circuits targeting strength, flexibility, balance, and muscular endurance—dimensions that are essential for maintaining independence in patients with chronic conditions. Furthermore, incorporating muscular training into SL projects offers physiotherapy students a deeper understanding of the biomechanical and physiological demands of rehabilitation. By engaging in planning and executing interventions aimed at improving neuromuscular function, students become familiar with core elements of clinical exercise prescription, including load progression, activation of stabilizing muscle groups, and modulation of intensity based on patient tolerance. The musculoskeletal system serves as the foundation for therapeutic recovery across various clinical scenarios. Training students to evaluate and address muscle imbalances, postural dysfunctions, and muscular deconditioning within real-life environments prepares them for evidence-based clinical practice. These educational experiences promote the development of not only academic knowledge but also manual assessment skills, functional diagnosis, and the ability to translate muscular outcomes into patient-centered goals.

While the pedagogical benefits of SL are well-documented, few studies have applied this methodology to therapeutic exercise targeting cardiorespiratory conditions in community settings.

Therefore, this pilot study aims to explore the implementation of Service-Learning in physiotherapy education, with a focus on the use of public community spaces for the design and execution of therapeutic exercise programs targeting cardiorespiratory conditions. Specifically, it examines students’ acquisition of technical knowledge and the development of key professional competencies such as clinical reasoning, communication, adaptability, and teamwork. It explores the integration of SL methodology into therapeutic exercise planning in real community spaces—a topic that remains under-represented in the current literature.

## 2. Methods

A convergent mixed-methods design was selected to simultaneously collect and analyze quantitative and qualitative data, offering a comprehensive view of students’ perceived learning outcomes. The project was conducted in accordance with the ethical principles of the Declaration of Helsinki (2013 revision), and all participants were informed of the study objectives and gave informed consent prior to participation.

The sample consisted of 44 third-year students from the Physiotherapy Degree Program at the University of Extremadura. Students were organized into eight work groups of 5 to 6 members each and were assigned clinical case studies involving cardiorespiratory pathologies. Four groups focused on respiratory conditions—specifically chronic obstructive pulmonary disease (COPD) and pulmonary fibrosis—while the remaining four groups addressed cardiac pathologies, namely acute myocardial infarction and ischemic heart disease.

The intervention was structured in five key stages: a theoretical phase, practical phase, community coordination, evaluation, and data analysis.

### 2.1. Theoretical Phase

In the initial phase, each group prepared a comprehensive report analyzing their assigned clinical case and proposing a tailored therapeutic exercise program. The report included the pathophysiology of the disease, clinical evaluation of the patient, rationale for the selected exercises, and a detailed plan that considered dosage, frequency, intensity, and other implementation variables. Students were also required to conduct a literature review on SL in physiotherapy contexts to justify their proposals. All activities were supervised and guided by two faculty physiotherapists.

This stage focused on developing key competencies such as evidence-based clinical reasoning, planning of therapeutic interventions, and written communication of clinical content.

### 2.2. Practical Phase

**Figure 1:**
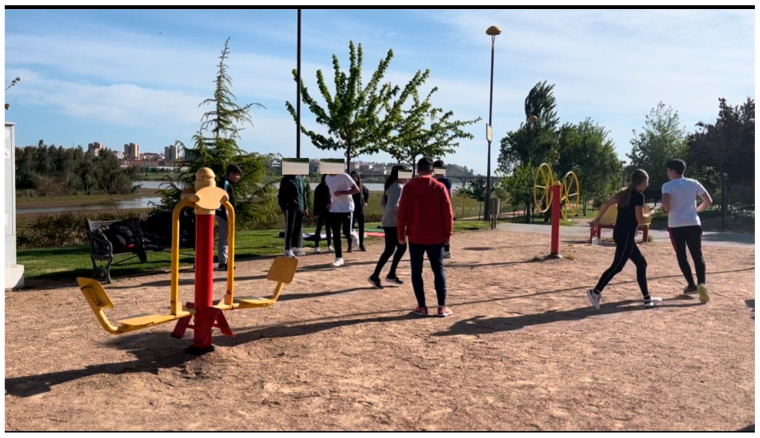
Students applying the exercise program at the bio-healthy park.

The proposed programs were implemented in real community environments through role-playing exercises. Students alternated between the roles of physiotherapist and patient to simulate intervention delivery and assess feasibility. Adjustments were made based on spatial accessibility and individualized patient needs. Therapeutic exercise circuits were designed and conducted in parks and sports centers, focusing on mobility, balance, aerobic capacity, and strength. The intervention followed an interval training format composed of three phases: warm-up, main activity, and cool-down. Students had to structure the exercise program with precise attention to periodicity, volume, intensity, number of repetitions, and physiological control variables—tailored to the assigned pathology (COPD, pulmonary fibrosis, myocardial infarction, or ischemic heart disease). Functional assessment tools (e.g., 6 min walk test, Borg scale) were used to monitor response and inform exercise adaptation. All sessions were supervised by teaching staff (Figure 1).

This phase emphasized the acquisition of competencies such as clinical decision-making, therapeutic adaptation, and functional assessment in community settings. Particular attention was given to the activation and progression of major muscle groups involved in postural support, locomotion, and cardiorespiratory efficiency. Exercises were designed to target the lower limb extensors, core stabilizers, scapular muscles, and respiratory musculature, which are essential for maintaining independence and functional capacity in patients with chronic cardiovascular and pulmonary conditions.

Students were encouraged to apply principles of progressive overload, neuromuscular control, and compensatory training when adjusting exercise variables. This not only reinforced biomechanical concepts, but also deepened their understanding of muscle physiology, adaptation mechanisms, and fatigue thresholds in clinical populations.

### 2.3. Coordination with the Community

Collaboration with healthcare professionals and community stakeholders was integral to ensuring the contextual viability and sustainability of the intervention. Meetings were held with physiotherapists, physicians, and patient associations to review the exercise proposals and evaluate the suitability of public spaces. These interactions aimed at aligning the project with community needs and foster engagement with local health networks.

Key competencies addressed in this phase included interprofessional collaboration, community health engagement, and project feasibility analysis.

### 2.4. Evaluation

To evaluate the impact of the project, three evaluation strategies were implemented: self-/co-evaluation using rubrics, satisfaction questionnaires, and teacher observation. The variables assessed included the following: acquisition of theoretical knowledge, development of communication skills, problem-solving in real environments, and adaptability to non-clinical contexts. In addition, students completed reflective diaries in which they critically evaluated their own learning, challenges faced, and the applicability of SL to future clinical practice. The competencies assessed included the following: (1) clinical reasoning, (2) patient communication, (3) adaptability to context, and (4) teamwork. Each was operationalized through specific indicators within the rubric.

This combination of tools allowed for a multidimensional evaluation of both learning outcomes and professional development.

The instruments used for evaluation included self-assessment and peer-assessment rubrics adapted from previous validated educational studies [17]. While some tools were custom-designed for this context, their structure was based on established competency frameworks used in health sciences education, and included domains such as clinical reasoning, communication, and adaptability. All instruments were reviewed by two expert faculty members to ensure content validity.

### 2.5. Data Analysis

A descriptive statistical analysis was carried out using SPSS software (version 23, IBM Corp.), including frequencies, means, and standard deviations. No inferential tests were applied, as no group comparisons or pre/post measures were involved.

The analysis focused on evaluating students’ perceptions of their learning outcomes and the implementation of the Service-Learning (SL) experience.

A mixed-methods analysis was conducted. We calculated frequencies, percentages, means, and standard deviations for the data collected through self-assessment rubrics, peer-assessment rubrics, and satisfaction questionnaires. This approach was appropriate given the exploratory nature of the study, which did not include a control group or pre-intervention measurements. The aim was not to establish statistical differences or infer causality, but to identify general trends in the students’ responses and perceived development of competencies. No inferential statistical tests were applied.

Qualitative data from reflective writings and open-ended responses were analyzed thematically, identifying recurring categories and key patterns in student experiences. Student reflections were analyzed through thematic content analysis. Two researchers independently coded the data, identified recurring themes, and reached a consensus through iterative discussion to ensure trustworthiness.

This methodology provided a comprehensive understanding of the educational impact of SL, while also identifying practical opportunities for implementing therapeutic exercise programs in real community settings. These findings are expected to support future student-led interventions with real patients.

## 3. Results

The study yielded significant outcomes in three key areas: acquisition of theoretical knowledge, development of transversal competencies, and student perception of the Service-Learning (SL) experience.

**Figure 2:**
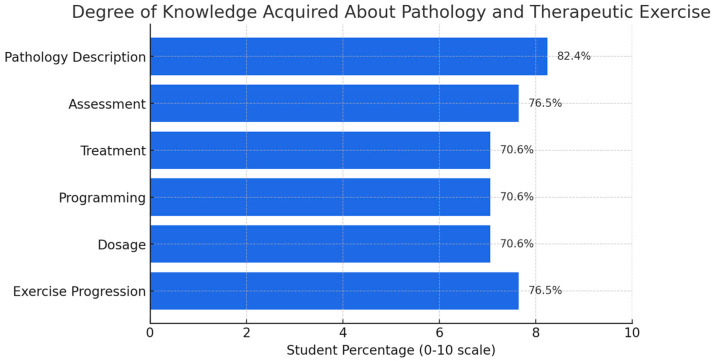
Student self-assessment results on knowledge acquisition related to cardiorespiratory pathologies (N = 44). Ratings are based on a 0–10 scale. Higher scores reflect greater perceived learning in clinical description, assessment, and therapeutic exercise planning.

Students demonstrated a high level of understanding regarding cardiorespiratory pathologies and therapeutic exercise. Quantitative results revealed strong performance in core learning dimensions, particularly in the accurate description of clinical conditions, patient assessment, and the design of individualized exercise programs. More than 70% of participants rated their learning outcomes between 8 and 10 (on a scale from 0 to 10) across these variables (Figure 2). These findings reflect a solid assimilation of clinical content and a growing ability to apply theoretical knowledge to real-world contexts, particularly within community-based rehabilitation settings.

In addition to cognitive learning, students reported notable improvements in several transversal competencies. Among the most highly rated were the following: teamwork, positively evaluated by 92% of participants; adaptability, with 89% reporting enhanced ability to respond to changing environments and patient needs; and decision-making, highlighted by 87% of participants as a skill developed through the intervention process.

Furthermore, 94.1% of students considered the use of cooperative learning methodologies in community spaces to be beneficial to their professional training. All participants (100%) positively valued the inclusion of SL projects as part of their physiotherapy education, reinforcing the perceived relevance and impact of this pedagogical model.

Thematic analysis of student reflections revealed several recurring themes. One student wrote the following: “*I was able to see how my proposal could be applied in real life and realized how important it is to adapt to the available space and the patient’s limitations.*” Another commented: “*The most valuable part was working as a team and taking on the role of a physiotherapist in a setting that simulates real-life practice*”. These excerpts illustrate the perceived impact of SL on the students’ sense of professional readiness and engagement.

The evaluation strategies used—namely self-assessment, peer (co-)assessment, and teacher observation—converged in their results. Self-assessment yielded an average score of 9.18/10, while co-assessment resulted in a slightly lower but consistent score of 9.04/10, indicating a shared and realistic perception of skill development and knowledge acquisition among the students.

Faculty observations further supported these outcomes, emphasizing the students’ ability to effectively translate theoretical content into practical applications. Teachers also noted a high degree of engagement with the SL methodology and a strong commitment to working in real-world, community-based contexts.

Additionally, students demonstrated increased awareness of the role of muscle function in physiotherapy interventions. The focus on functional muscle performance in diverse community settings appeared to foster students’ ability to assess, adjust, and prescribe exercises aligned with patient-specific muscular demands—an essential competency in physiotherapy practice.

## 4. Discussion

This pilot study suggests that Service-Learning may be a promising educational strategy, particularly in promoting students’ engagement and perceived competency development [2,3,5]. The high ratings related to knowledge acquisition in cardiorespiratory physiotherapy suggest that learning in real or simulated community environments enhances the assimilation of clinical content and promotes effective transfer of knowledge to practice [15,22]. These results align with those of Borstad et al. [16], who observed improved theoretical understanding and practical application in students participating in SL projects.

Beyond cognitive gains, the development of transversal competencies—such as teamwork, adaptability, and decision-making—emerged as a key benefit of the SL experience. The literature highlights the fact that community engagement fosters empathy, civic responsibility, and critical thinking [23,24]. Our findings are consistent with these observations, particularly regarding communication, collaboration, and leadership skills [2,24]. Students also reported increased awareness of the social role of physiotherapists and a deeper sense of responsibility toward vulnerable populations [3,5].

The high levels of student satisfaction reinforce the motivational value of this pedagogical approach, echoing prior studies that link SL with greater engagement and academic performance [4,22]. However, previous research also indicates that the success of SL projects depends significantly on the quality of faculty supervision and the structure of student involvement [25], underlining the need for well-planned and clearly defined project frameworks [9].

Students positively evaluated teamwork (with scores above 7 in most cases), and 94.1% of them considered the integration of cooperative methodologies and community resources to be beneficial for their professional training. The alignment of scores in self-assessment (mean = 9.18/10) and peer-assessment (mean = 9.04/10) indicates the reliability of these tools in evaluating learning outcomes. These tools also promote reflective learning. Nonetheless, the implementation of more detailed indicators within assessment rubrics could further improve the objectivity and precision of future evaluations. The literature supports the use of structured evaluations complemented by feedback from faculty and community stakeholders to provide a more holistic assessment of student learning [26].

### 4.1. Limitations and Future Lines

This study encountered several limitations. First, there were logistical difficulties in coordinating with community organizations posed challenges, particularly in identifying suitable and accessible intervention sites. These issues are well documented in the literature, where authors such as Tijsma et al. [26] emphasize the importance of sustainable and mutually beneficial partnerships in SL initiatives.

Second, the project was conducted in a simulated environment without the participation of real patients, which may have influenced students’ perceptions of the applicability of their proposed interventions. Although the exploration of real environments allowed students to assess conditions and resources, additional challenges may arise when working with actual patients. Prior studies suggest that SL projects involving real patients tend to foster deeper empathy and enhance the students’ understanding of patient needs [17,18]. While this allows for structured training and experiential learning, it limits the external validity and real-world clinical transferability of the results.

On the other hand, the sample was limited to a single academic institution and cohort, which may affect the generalizability of the findings. Third, although qualitative reflections added depth to the analysis, the absence of triangulation with patient outcomes or long-term follow-up data may reduce the comprehensiveness of the evaluation.

Finally, given the absence of pre-intervention measurements and a control group, the study does not aim to establish causal relationships. Instead, it explores perceived educational outcomes from the students’ perspective within a real-world community context. These findings should be interpreted as preliminary, pending further studies using pre/post designs and validated outcome measures.

Future iterations of this project should consider expanding the experience to real clinical contexts through partnerships with healthcare providers and patient associations. This would strengthen the clinical relevance of the training and further bridge the gap between academic education and professional practice.

### 4.2. Impact on Student Education, Teacher Perception and Applicability of the Methodology

The SL experience promoted the development of essential professional competencies, including clinical decision-making, adaptability, and leadership. Students reported that working in real environments and engaging with community resources enhanced their ability to design and implement meaningful, patient-centered therapeutic exercise programs. Furthermore, the process of evaluating the feasibility of their interventions encouraged critical reflection on the alignment of proposed treatments with real-world needs.

From the perspective of faculty, SL proved to be a highly effective educational strategy. Teachers observed strong student engagement, motivation, and responsibility throughout the experience. The simulation of real-world interventions prior to direct contact with patients also enabled students to identify and correct planning errors, thus reinforcing their practical and reflective skills.

### 4.3. Project Projection and Continuity

Based on the positive results obtained, the extension of the project to include real patient interventions is strongly recommended. This would involve collaborations with patient associations, healthcare professionals, and municipal organizations. In addition, the development of educational materials and dissemination strategies could enhance the social visibility and long-term sustainability of the initiative.

Overall, this experience reaffirms SL as a powerful pedagogical approach in physiotherapy education, contributing not only to technical and clinical skill development but also to the formation of socially conscious, community-oriented health professionals

In future phases, it will also be essential to deepen the integration of muscle-specific objectives into student-led rehabilitation strategies, ensuring alignment with clinical guidelines for musculoskeletal recovery in chronic cardiorespiratory conditions.

On the other hand, the results of this pilot study support the potential scalability of this Service-Learning (SL) protocol to broader contexts. Future developments could focus on the adaptation of this methodology to international educational environments, considering cultural and infrastructural differences while preserving the core focus on musculoskeletal competency development in physiotherapy students. The protocol may serve as a framework for integrating muscle-focused therapeutic exercise training in undergraduate curricula across different countries. With appropriate contextual adjustments, similar SL experiences could be implemented to promote the acquisition of clinical reasoning, therapeutic planning, and functional rehabilitation skills targeting musculoskeletal health. Collaborative networks among physiotherapy education institutions could facilitate the cross-cultural validation of this SL-based approach, supporting its incorporation into international standards for physiotherapy training in musculoskeletal rehabilitation.

### 4.4. Muscular Focus in Therapeutic Exercise

A key aspect of the intervention was the design of exercise programs targeting muscular performance, particularly of the lower limbs, trunk stabilizers, and postural control systems. The functional circuits implemented in parks and public spaces included strength exercises (e.g., bodyweight squats, stair climbing, and resistance band routines), aerobic walking protocols, and flexibility routines aimed at improving joint mobility. These were not only oriented toward enhancing cardiorespiratory capacity but also to improving muscular strength, endurance, and neuromuscular coordination, all of which are key components in the rehabilitation of individuals with chronic cardiorespiratory conditions.

The importance of muscle function in the context of chronic diseases such as COPD and heart failure is well established. Skeletal muscle dysfunction, particularly in the quadriceps, is one of the main extrapulmonary manifestations of COPD and has been associated with reduced exercise tolerance and quality of life [27,28]. Similarly, patients with heart failure often experience decreased muscle mass and function, which contributes to fatigue and limited physical activity; resistance training has been shown to counteract these effects and improve functional capacity [29].

Through this Service-Learning experience, students became familiar with the role of therapeutic exercise in promoting muscular adaptations in clinical populations. They learned how to select appropriate exercises to stimulate targeted muscle groups, tailor intensity and progression to individual limitations, and adapt their interventions to non-traditional settings. The community-based nature of the intervention challenged students to creatively adjust their programs to the available space and equipment, emphasizing bodyweight and functional training over machine-based protocols. These strategies reflect a patient-centered and functionally relevant approach to rehabilitation, particularly in settings with limited resources. Moreover, students reported gaining confidence in identifying muscular priorities during assessment, such as distinguishing between endurance deficits versus strength limitations, and understanding how to adapt exercise plans accordingly.

This practical focus contributed to students’ understanding of the musculoskeletal system’s central role in functional recovery. It also reinforced the importance of prescribing exercise with clear muscular objectives, such as improving lower limb strength to facilitate walking or enhancing trunk stability to support respiratory mechanics. These insights align with current physiotherapy practice, highlighting the integral role of muscle rehabilitation within cardiorespiratory physiotherapy and reinforcing the need for educational programs that emphasize musculoskeletal competence as a foundation for functional recovery.

## 5. Conclusions

This pilot study provides preliminary evidence that Service-Learning (SL) may be a valuable educational strategy in physiotherapy training, particularly in promoting student engagement and perceived development of professional competencies such as teamwork, communication, and clinical reasoning.

The findings suggest that integrating SL into therapeutic exercise planning in community environments is well received by students and may enhance their ability to connect theoretical knowledge with practical application. However, these conclusions are based on students’ subjective perceptions, not on objective learning outcomes or validated performance measures.

Several limitations must be acknowledged: the absence of pre- and post-intervention assessments, the lack of a control group, the exclusive reliance on self-reported data, and the absence of real patient involvement. These factors limit the generalizability and interpretability of the findings. Future research should aim to implement controlled designs, include real clinical scenarios, and incorporate validated tools to measure both learning outcomes and clinical effectiveness. Nonetheless, the present study lays the groundwork for future educational initiatives that seek to integrate community-based practice with student learning in physiotherapy education.

Service-Learning is not only a means of academic development, but a transformative educational strategy that bridges the gap between university and society. The focus on muscular function in therapeutic planning, particularly in community settings, provides an opportunity to align physiotherapy education with real-world rehabilitation needs. By engaging students in designing exercise programs targeting muscle strength, control, and endurance, this model enhances their readiness to contribute to the promotion of musculoskeletal health in both clinical and preventive contexts.

## Data Availability

Data are contained within the article.
